# Specific age related signatures in Drosophila body parts transcriptome

**DOI:** 10.1186/1471-2164-7-69

**Published:** 2006-04-04

**Authors:** Fabrice Girardot, Christelle Lasbleiz, Véronique Monnier, Hervé Tricoire

**Affiliations:** 1 Biologie du Développement, UMR7009 CNRS/UPMC, Observatoire Océanologique, Quai de la Darse, 06234 Villefranche-sur-Mer Cedex, France; 2 Département de développement, Institut Jacques Monod, 2 place Jussieu, 75251 Paris, France

## Abstract

**Background:**

During the last two decades progress in the genetics of aging in invertebrate models such as *C. elegans *and *D. melanogaster *has clearly demonstrated the existence of regulatory pathways that control the rate of aging in these organisms, such as the insulin-like pathway, the Jun kinase pathway and the Sir2 deacetylase pathway. Moreover, it was rapidly shown that some of these pathways are conserved from yeast to humans.

In parallel to genetic studies, genomic expression approches have given us significant information on the gene expression modifications that occur during aging either in wild type or long-lived mutant animals. But most of the genomic studies of invertebrate models have been performed so far on whole animals, while several recent studies in mammals have shown that the effects of aging are tissue specific.

**Results:**

We used oligonucleotide microarrays to address the specificities of transcriptional responses in aging Drosophila in head, thorax or whole body. These fly parts are enriched in transcripts that represent different and complementary sets of genes. We present evidence for both specific and common transcriptional responses during the aging process in these tissues. About half of the genes described as downregulated with age are linked to reproduction and enriched in gonads. Greater downregulation of mitochondrial genes, activation of the JNK pathway and upregulation of proteasome subunits in the thorax of aged flies all suggest that muscle may be particularly sensitive to aging. Simultaneous age-related impairment of synaptic transmission gene expression is observed in fly heads. In addition, a detailed comparison with other microarray data indicates that in aged flies there are significant deviations from the canonical responses to oxidative stress and immune stress.

**Conclusion:**

Our data demonstrates the advantages and value of regionalized and comparative analysis of gene expression in aging animals. Adding to the age-regulated genes already identified in whole animal studies, it provides lists of new regionalized genes to be studied for their functional role in the aging process. This work also emphasizes the need for such experiments to reveal in greater detail the consequences of the transcriptional modifications induced by aging regulatory pathways.

## Background

For many years the aging process has been a major subject of interest for biologists because of its complexity and diversity: the lifespans of closely apparented species can be very different, and there are species such as turtles that do not seem to age at all. In addition, the large number of phenotypic features that are modified by aging such as fertility, mobility and memory, illustrates the variety of organs and tissues that are affected by the aging process.

Although many theories have tried to explain aging, only few experimental advances were made prior to the last two decades. Since then rapid progress in the genetics of aging has been made in invertebrate models such as *C. elegans *and *D. melanogaster*, demonstrating the existence of regulatory pathways that control the rate of aging in these organisms [1-14]. They include the insulin-like pathway, the Jun kinase pathway and the Sir2 deacetylase pathway. Moreover, it was rapidly shown that some of these pathways are conserved from yeast to humans.

In parallel to genetic studies, genomic expression studies have brought significant information on the gene expression modifications occurring during aging either in wild type or long lived mutated animals. Several groups have demonstrated a strong correlation between patterns of aging and those observed during the oxidative stress response. Microarray studies of *C. elegans daf2 *and *daf16 *mutated animals confirmed the importance of the genes involved in stress protection for the control of lifespan by the Insulin/IGF1 pathway [15-17]. Together with subsequent functional RNAi studies these studies also pointed out the importance of other features controlled by these pathways, notably the regulation of genes involved in mitochondrial function and fat metabolism [18].

Until now most of the genomic studies of invertebrate models have been performed on whole animals. Several studies, however, recently performed on specialized mammalian tissues, either post-mitotic (heart or nervous system) or mitotic (liver), show that the effects of aging are tissue-specific [19-25]. In addition, effects of caloric restriction on age related transcriptional changes are also tissue- or species-specific [19].

To better understand the aging process in invertebrate models it is thus highly desirable to investigate transcriptional changes at the tissue level. Because of the small size of the animals involved (nematode and drosophila) microarray studies on purified tissues represent a technical challenge. Nevertheless, one would expect that studies of body parts of these animals which are greatly enriched in specialized tissues would bring useful information. In Drosophila the head, enriched in neuronal tissue with minor contributions from fat and muscles, and the thorax, enriched in muscle with contributions from nervous and digestive systems, provide good opportunities to study age related regionalized transcriptional changes. A first step in this direction was taken recently with studies of gene expression in Drosophila head [26]. Nevertheless, this study was not sufficiently extensive since it was performed on chips including only one third of the genome. Moreover, it was performed by mixing male and female heads, which could be a source of confusion.

In this paper we present data obtained on Affymetrix chips for young (3 days old) and old (40 days old) flies. We focussed on the head and thoraces since transcription in brain and muscles have been shown to be strongly affected by aging in mammalian studies. We have simultaneously analyzed gene expression in the head, the thorax and whole flies. We present evidence for both common and specific responses in these body parts and identify new genes and processes that are altered in aging flies, which could not be identified previously on whole fly experiments. Greater downregulation of mitochondrial genes and activation of JNK pathway in the thorax of aged flies suggest that muscle may be particularly sensitive to aging. Conversely, age related transcriptional changes observed in the head suggest that there is strong impairment in synaptic transmission during the aging process. In addition, using complementary published data, we show that many of the genes described as downregulated with age are linked to reproduction and overexpressed in gonads. Our data demonstrate the relevance of regionalized analysis of gene expression and emphasizes the need for such experiments to expose in more detail the consequences of transcriptional modifications induced by aging regulatory pathways.

## Results

### A large fraction of age downregulated genes are sex specific and gonad biaised

To compare transcriptional modifications occurring in different Drosophila body parts during aging and to compare these data with previous observations obtained on whole flies, batches of 3 day- and 40 day-old male flies, which underwent the same rearing conditions, were used for RNA preparations from whole flies, heads or thoraces. Importantly, the same flies were used for preparation of heads and thoraces, thus minimizing spurious variations. Comparisons were performed either between body parts at both ages or between old and young flies for each body part and the data were processed as described in Material and Methods. A file including the mean values and standard errors for the different pairwise comparisons for all the probe sets, a summary of the probe sets associated to statistically significant variations and the cluster identifications used in the following of this analysis are provided as Additional file 1.

**Figure 1:**
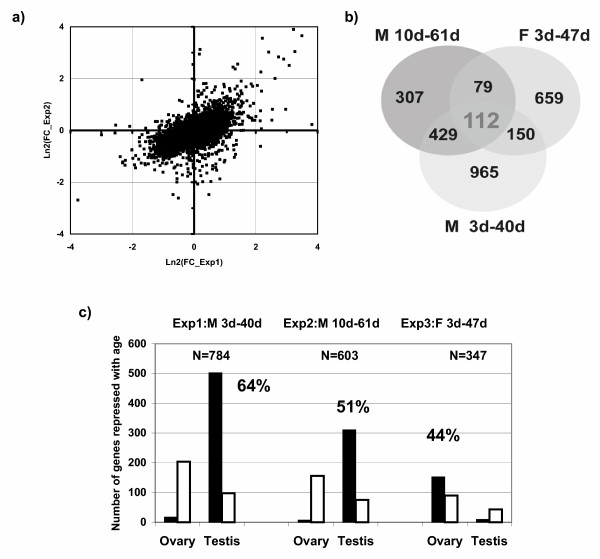
**Comparative analysis of aging experiments**. We compare our data (Exp. 1) to microarray data obtained on male flies from [27] (Exp. 2) and on female flies [28] (Exp. 3). a) Correlations between the experiments performed on males. The reported fold change (FC) corresponds to 3- and 40-day old males of (Exp1) and 10- and 61 day-old males of (Exp2). The correlation coefficient between the two sets of data is 0.6. b) Venn diagram of the number of probe sets showing significant age-related changes in the three experiments. 112 probesets were identified as age-responsive in the three conditions. c) Repartition of the age downregulated probe sets (ADP) in ovary or testis biased classes according to data from [29]. The total number of ADP is indicated for each experiment. The white bars represent the distribution expected from a distribution of the ADP similar to that observed at the genome level (26% ovary biased and 16% testis biased). The black bars represent the observed numbers with the corresponding percentage of total ADP. Notice the significant enrichment of the ADP in gonad enriched genes for both sexes.

Among the 8760 probe sets detectable on the Affymetrix chip, 2760 (32%) display a significant variation between 3 day- and 40 day-old flies in at least one comparison (whole body, head or thorax). Only 1656 probe sets (19%) show significant variations with aging in whole body male flies. A good agreement was observed between our data and results from [27]: 58% of the genes identified as age-responsive in this latter experiment were also detected in our work and we observed a significant correlation between the fold changes observed during aging of male flies in the two experiments (Fig. 1a). The remaining discrepancies may arise from various causes, such as differences in fly strains, differences in fold change threshold or, more likely, from differences in the age of analyzed flies (3 days and 40 days in this work, 10 days and 61 days in [27]).

In contrast to this good correlation between experiments performed with male flies we observed a poorer correlation with previous data obtained on aging female flies [28]: among the 1000 probe sets defined as age-responsive in this latter study and present in our chips only 262 (26%) showed significant transcriptional changes in our experiment, while this proportion lower to 19% with data from Landis et al. (Fig. 1b). Consequently only 112 probe sets were detected as age-responsive in the three experiments (see list in Additional file 4). Subsequent observations showed that this poor correlation is mostly due to age repressed genes that are largely specific in male or female experiments. Indeed, a likely explanation is that these transcripts are gonad specific transcripts that could be repressed during the aging process. Therefore, to test this hypothesis, we used data from Parisi et al. [29], who performed a large number of comparisons between different genotypes and dissected tissues to identify ovary, testis and soma biased transcripts. This enabled us to perform a more detailed analysis of the genes identified as age repressed in the three aging experiments (Additional file 1). Compared to the expected distribution of ovary (26%) or testis (12%) biased transcripts from the whole genome results of Parisi et al., the genes repressed with age in male flies are strongly enriched (p < 10^-13^) in testis biased genes (Fig. 1c). As many as 64% (this work) and 51% [27] of the genes downregulated in aged Drosophila males are testis biased. Conversely, genes repressed in aged female flies are strongly enriched (p < 10^-12^) in ovary biased genes (Fig. 1c). Overall, our results suggest that about half of the genes repressed during the aging process in both sexes are gonad biased. This repression of gonad genes correlates to the decrease in reproduction observed during aging. This result emphasized the need for a more detailed analysis of tissue-specific transcriptional variations during aging and prompted us to investigate, in a first step, age related transcriptional changes in different body parts.

### Identification of head and thorax enriched transcripts

**Figure 2:**
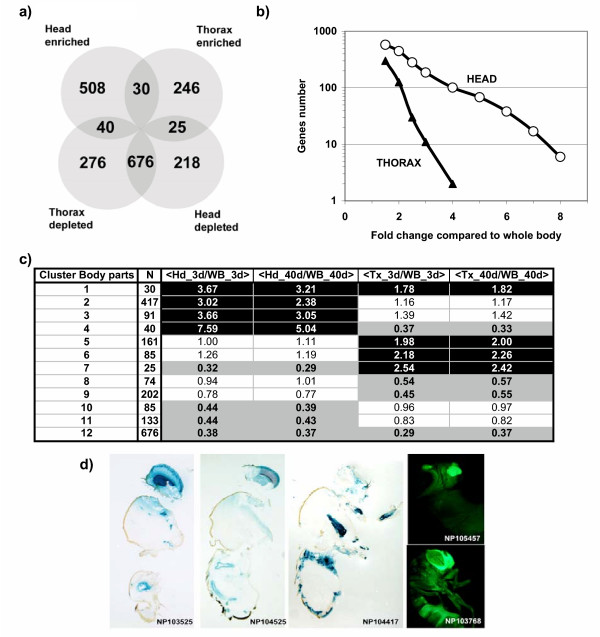
**Analysis of body part gene specificity**. a) Schematic distribution in a Venn diagram of the 2019 probe sets presenting a statistically significant enrichment or depletion in head or thorax compared to whole body after SAM analysis with a fold changeof 1.5. b) Evolution of the number of genes enriched in head or thorax with increasing fold change compared to whole body. c) Clustering of the responsive probesets. The first and second columns contain the cluster number for body part analysis and the number of probe sets in each cluster, respectively. The last four columns contain the mean value of the fold change compared to whole body for all the members of the cluster for the four different conditions (head or thorax at 3 or 40 days). Black or gray areas emphasize significant positive (enrichment) or negative (depletion) fold changes, respectively. Note that since the cluster 12 contains a large number of probe sets (676) that are depleted in both head and thorax compared to whole body, it should therefore be interpreted as containing genes enriched in the fly abdomen. d) Exemples of tissue specific UAS-LacZ or UAS-GFP expression driven by GAL4 enhancer trap insertions located inside the regulatory regions of head or thorax enriched genes. The corresponding genes are the head enriched genes SoxN (NP103525), spir (NP104325), CdsA (NP103768), CG31241 (NP105457) and the thorax enriched gene CG9572 (NP104417). In the latter two cases these expression data may give some indication of the role of these genes of unknown function.

First, we identified transcripts either enriched or depleted in body parts compared to whole body. To minimize false positives we considered only probe sets that presented similar statistically significant variations (Fold change >1,5; FDR<1%) for both time points (3 days and 40 days). Among the 8760 detectable probe sets on the Affymetrix chip, 2019 (23%) presented significant variations between adult male body parts (Fig. 2a). Subsequent analysis based on Boolean clustering allowed us to define 12 clusters with specific expressions in body parts (Fig 2c and Additional file 1 for complete list).

Significantly, we observed many more genes strongly enriched (FC>3) in the head (N = 186), that contains large specialized structures such as the eye or the brain, than in the thorax (N = 11) (Fig. 2b). The list of the most head- or thorax-enriched probe sets is provided in Additional file 2. Using the Gene Ontology database, we found that genes associated with transmission of nerve impulse, organogenesis, response to abiotic stimuli (including radiation), signal transduction activity, ion channel activity and calmodulin binding are strongly over-represented (p < 10^-3^) in the genes identified as head enriched (see Additional file 3 for complete analysis). This set includes a large number of eye-specific genes (norpA, inaC, inaF, rhodopsines,...) as well as genes encoding proteins involved in neuronal or glial functions (Choline acetyltransferase, histidine decarboxylase, muscarinic Acetylcholine Receptor 60C, excitatory amino acid transporter 2, ...).

The signatures of genes associated with the thorax enriched probe sets are strikingly different with over-representation in two classes of biological processes (p < 10^-4^), mesoderm development and organogenesis and two functional classes (p < 10^-3^), structural constituent of cytoskeleton and cytoskeleton protein binding. Inside these classes many genes involved in muscle development and/or muscle function can be readily identified such as *bent*, *Rya-r44F *(the ryanodyne receptor), *Msp-300 *(the muscle-specific protein 300), *tungus *and *vestigial*. However, almost half of the head- or thorax-enriched probe sets were not associated to a functional annotation.

We recovered a few enhancer trap lines where the GAL4 transposon is inserted inside the regulatory regions of head or thorax enriched genes. As expected, in many cases, these GAL4 lines were able to drive UAS-LacZ or UAS-GFP expression in a tissue-specific manner (Fig. 1d). In summary, and in agreement with our expectations, the head and thorax enriched transcripts represent different and complementary sets of genes, which can be studied for their expression during the aging process.

### Functional analysis at whole body level confirms relationships between mitochondrial dysfunction, stress response and aging

**Figure 3:**
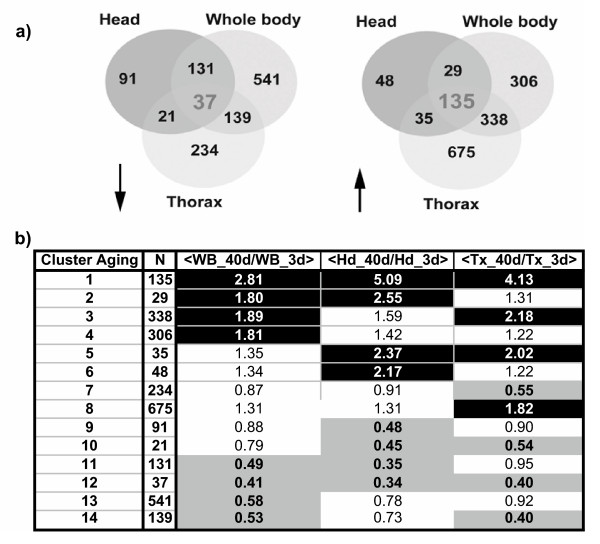
**Analysis of age dependent changes in differentbody parts**. a) Venn diagram of probe sets downregulated (left) or upregulated (right) with age in whole body, head or thorax. 37 downregulated and 135 upregulated probesets are common to all the tissues. b) Clustering of the responsive probe sets. The first and second columns contain the cluster number for aging analysis and the number of probe sets in each cluster, respectively. The last three columns contain the mean value of the fold change between 40-day old and 3-day old flies for all the members of the cluster in the three conditions (whole body, head or thorax). Black or gray are as emphasize significant positive (enrichment) or negative (depletion) fold changes, respectively.

A second step of data analysis allowed us to identify transcripts statistically upregulated or downregulated in different body parts as a function of age. To assess the statistical significance of the results, we used similar conditions to those described previously (FC >1,5; FDR<1%). From the 2760 probe sets that present significant variations with age in either body parts or in whole flies (Fig 3a) we defined, on the basis of a Boolean clustering, 14 clusters with specific age-dependent expressions in body parts (Fig. 3b and Additional file 1 for complete list). Interestingly, 1104 age-responsive probe sets (40%, clusters 5 to 10) were not detected in the whole fly analysis and are thus body part specific. In contrast, 37 age downregulated probe sets (1,3%, cluster 12) and 135 upregulated probe sets (4,9%, cluster 1) were identified in all conditions and thus represent a core response of the aging process.

Using the Gene Ontology (GO) annotation, we identified the molecular functions and biological processes that are over- or under-represented in the 1656 whole body age-responsive probe sets identified in our study compared to the distribution found for the complete set of 8760 detectable probe sets (see Additional file 3, whichgroups all the functional analyses performed for this manuscript). As expected from the results concerning gonad specific genes (see above), we found that genes associated with mating behaviour are significantly enriched (p < 0.002) in the age downregulated set. This latter group is also enriched in genes associated with oxidative phosphorylation, tricarboxylic acid cycle (TCA) and muscle contraction. In contrast, genes associated with the immune response and with amino acid metabolism are clearly enriched in the age upregulated set. Significantly, except for the mating behaviour process, all these features were conserved when we analyzed in a similar way the core of 112 probe sets (list in Additional file 4), which were identified as age-responsive in the 3 different experiments performed with either males or females.

In agreement with previous finding, we also noticed a significant correlation between stress-responsive genes and age-responsive genes: 21% of the whole body age-responsive probe sets identified in our study were also identified as responsive to paraquat, or H_2_O_2 _or tunicamycin in a previous work [30] (see Additional file 1 for multi cluster identification). A smaller number of these genes (12%) are also responsive to hyperoxia (data not shown).

**Figure 4:**
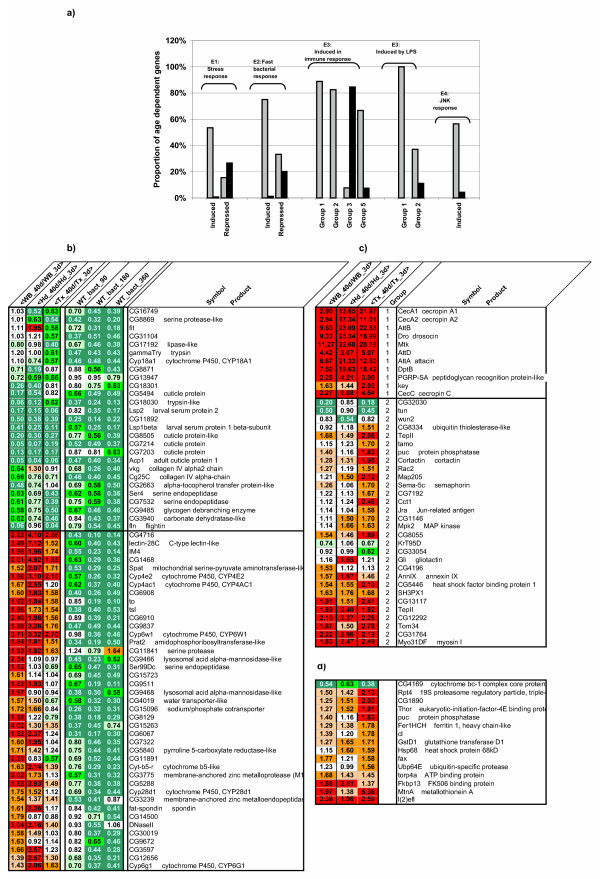
**Correlations between stress or immune responsive genes and age-responsive genes**. a) We analyzed the proportion of genes upregulated (grey bars) or downregulated (black bars) with age for sets of genes identified as stress- or immune-responsive genes in different large scale experiments: E1, microarray analysis of paraquat-induced oxidative stress response [30]; E2, microarray analysis of whole fly bacterial response before 360 h [34]; E3, microarray analysis of whole fly bacterial response and cellular inflammation response [32]; E4, JNK induced genes [12]. Abcissa labels refer to the classification of genes according to the data in each reference. Groups 1, 2, 3, 5 of immune response in E3 refer to genes from Imd/rel group, Toll group, cyto group and others groups in Table 1 of [32]. Note the good correlation between genes induced by stress (oxidative or immune) and genes overexpressed with age, and the poorer correlation between genes repressed by stress and age downregulated genes. In b) the age-responsive genes downregulated after bacterial immune challenge are reported with their fold change induced either by age or by immune stress. Note also the strong repression with age of immune responsive group 3 genes which could correspond to wound response genes. In c) and d) the LPS induced and JNK induced genes are reported with their fold change induced by age. Note in these two gene sets their stronger induction in the thorax compared to that observed in head or in whole fly. In particular note that *Jra *itself in induced in the thorax of old flies.

Nevertheless, in spite of the observed enrichment in immune response genes and stress-responsive genes, we noticed that the transcriptional signature of aging differs significantly (Fig. 4) from that described for these two process in previous experiments [27,30-33]. For instance, in paraquat induced oxidative stress, while 53% of the upregulated genes show similar variation during aging, stress downregulated genes were found in significant proportions, both in the age upregulated class (15%) and the age downregulated genes (27%) (Fig.4a, E1). A similar weak correlation in the direction of transcriptional changes was observed for the genes downregulated during immune response in [34] (Fig.4a, E2). The same observation can also be made by analysing the data of Landis et al[27]. Among the genes that vary in opposite directions in this latter case, one notes two lysosomal acid alpha mannosidase and five cytochrome P450 that could be used for detoxification (Fig. 4b). In addition we found that the group of early induced genes identified in bacterially infected flies [32] is strongly repressed during aging. All these features suggest that transcriptional changes during aging cannot be simply interpreted as being linked to the activation of stress response pathways (oxidative or immune). At least some aspects of these stress responses, such as activation of parallel branches of the pathways or a reduction in gene silencing, may be affected during aging.

### Aging signature in flies thoraces indicates an increased level of stress in aging muscles compared to other body parts

Among the 431 probe sets downregulated in the aged drosphila thoraxes, those associated to the cellular components muscle fibers and mitochondrial membranes are strongly over-represented (p < 5 10^-3^, see Additional file 3 for a detailed analysis). The latter are also mainly associated with two biological processes (cellular carbohydrate metabolism and generation of precursor metabolites and energy, including oxidative phosphorylation) and linked with several over-represented molecular functions: calcium ion binding, tropomyosin binding and microfilament motor activity, oxidoreductase activity, electron transport activity, including NADH deshydrogenase activity.

**Figure 5:**
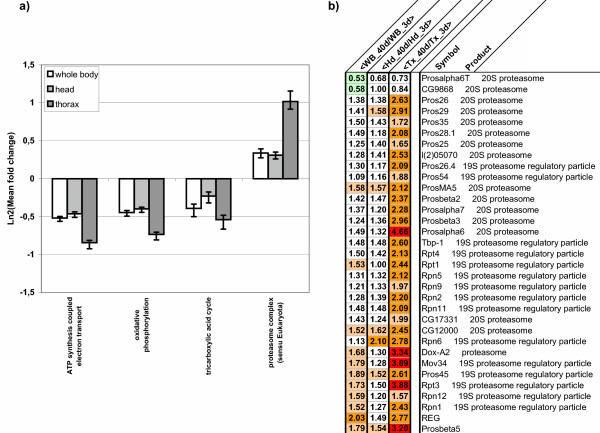
**Comparison of age related changes in body parts for some biological process**. a) For each probeset, we consider the differences in their fold changes between 40-day old and 3-day old flies for the three different conditions (whole body, head or thorax). The mean values for all the probesets associated with certain GO terms corresponding to biological processes were computed and plotted for the three conditions. Note that genes associated with oxidative phosphorylation (GO:0006119) and ATP synthesis (GO:0042773) are more strongly downregulated by age in the thorax (dark gray) than in the head (light gray) or at the whole body level (white). This is not observed for genes related to the TCA cycle (GO:0006121), which behave similarly in the three conditions. In contrast, the genes encoding members of the proteasome complex (GO:0000502) are much more strongly induced by age in the thorax than in the head or at the whole body level, as shown in more detail in b).

Among these 431 probe sets downregulated by age in the thorax, 234 were not found to be downregulated in the head or whole body. Interestingly, 3 genes associated with tropomyosin binding (*up*, *wupA *and s *pdo*) and a large number of genes associated with oxidative phosphorylation and ATP synthesis were found in these 234 probe sets. This suggests that mitochondrion function may be more greatly affected in aging muscles than in other aging tissues. Indeed, a closer examination of the age-related transcriptional changes in all the genes associated with these GO classes clearly revealed their increased downregulation in the thorax (Additional file 5 and Fig.5a): for instance, the mean fold change of all the genes related to ATP synthesis was 0.56 ± 0.03 in the thorax, compared to 0.70 ± 0.02 and 0.73 ± 0.02 in the whole body and head, respectively. In contrast, only marginal differences were observed for genes related to TCA (Fig. 5a). Interestingly, the mitochondrial MnSOD (*sod2*) is significantly downregulated in the aged thorax, which suggests that oxidative stress defenses are also lowered in the thorax compared to other body parts.

In upregulated probe sets in the aged thorax, we observed a significant over-representation (p < 5 10^-3^) in immune response genes, genes linked to cellular morphogenesis and actin filament based processes, as well as cellular components of the endoplasmic reticulum and of the proteasome complex (Fig. 5a, b). Interestingly, several features suggest that part of this response and notably the upregulation of genes associated with cellular morphogenesis may reflect the activation of the JNK pathway. Firstly, the Drosophila *Jun *homologue (*Jra*) is induced in the thorax of aged flies together with its representative target the phosphatase puckered (*puc*). Secondly a significant number (38%) of the genes known to be induced by LPS in a JNK-dependent manner in S2 cells [32] were also induced in the thorax (Fig. 4a, E3 and 4c). This group of genes includes several factors involved in cytoskeletal rearrangements such as the Cortactin, Myo31DF and Rac2. Thirdly, a significant number (N = 13, 57%) of stress genes responsive to JNK signaling identified by SAGE experiments in embryos or photoreceptor cells [12,13,35] are also age regulated (Fig. 4a, E4 and 4d), especially in the thorax.

All together, our data suggest that, compared with other body parts, the aged thorax undergoes an increase in mitochondrial impairment combined with a decrease in oxidative stress defenses, an activation of the JNK stress pathway and an upregulation of proteasome subunit transcription. Interestingly, this last feature was also observed in whole flies subjected to paraquat-induced oxidative stress [30].

### Genes involved in synaptic transmission are downregulated in heads of aged flies

A previous partial transcriptome analysis [26] identified "response to light" (GO:0009408) as the most prominent function associated with aged Drosophila heads. In agreement with these data, we also observed a significant downregulation of genes belonging to this GO class : the mean ratio between 40 day heads and 3 day heads was 0.84 and several genes implicated in rhodopsin mediated phototransduction (*ninaC, inaC, inaD, Arr1*) were repressed more than 1.5 times. The guanylate cyclase α subunit (*Gycα99B*) was the most age repressed gene of this class (3.7 times). In contrast the rhodopsin genes themselves did not present any significant age variations. This rules out the hypothesis that the global downregulation of genes involved in light perception may be caused by a general cellular misregulation in the photosensory organs. Interestingly, our data also imply that, in the aged head, calcium homeostasis and/or signalization may be perturbed since two calcium channels (*trp *and trp)l are downregulated.

On the other hand, in contrast to data from [26], the most significant (p < 2.10^-4^) aging signatures that we observed for head downregulated genes were enrichment in two classes: reproductive behaviour and transmission of nerve impulse. In the former class, 7 members of the accessory peptide family (Acp) and 3 members of the cluster of male specific transcripts Mst57D protein family that have been identified in adult head EST libraries, were present. Only the Acp36DE gene has been detected in [26] as downregulated, probably due to mixing of data from males and females. This indicates that, in addition to a general downregulation of gonad-enriched genes mentioned previously, aging downregulates some sex-linked genes in the head where their role is still elusive.

Head downregulated genes involved in synaptic transmission can be divided into three subgroups. The first one includes genes that play a role in neurotransmitter metabolism such as the choline acetyltransferase (*Cha*) and the dopamine N acetyltransferase (*Dat*) genes. They are both also downregulated in the thorax. Another member of this category, the Dopa decarboxylase gene (*Ddc*), is downregulated in whole flies and shows a similar trend in the head (but below our statistical significance threshold). In the second subgroup many genes involved in various steps of neurotransmitter secretion [36] appear to be affected: priming for synaptic vesicle fusion (*γ-SNAP, unc13, comatose *and *tomosyn*), fusion with presynaptic membrane (*Csp, Syx1A *and *rab3-GAP*) and reformation of vesicles through endocytosis (*liquid facets, AP-50 *and *AP-2σ*). Interestingly, most of these genes are strongly over-expressed in the head and do not present such an age related downregulation at the whole body level. The third subgroup includes several neurotransmitter receptor ion channels. Among these channels, two nitotinic acetylcholine receptors (*nAcRβ96A *and *nAcRα18C*) and three ionotropic glutamate receptors (*Nmdar1*, *GluCla *and *CG11155*) mediate excitatory synaptic transmission. Moreover, three inhibitory GABAergic channels (*Lcch3*, *GABA-B-R2 *and *Rdl*) are also downregulated in aged Drosophila heads. Additionally, the observed downregulation of the histamine-gated chloride channel subunit 1 (HisCl1), putatively involved in photoreceptor synaptic transmission, further strengthens the idea of an altered response to light in aged flies.

Upregulated genes in aged Drosophila heads mostly present signatures similar to those observed in whole flies (Additional file 3) : genes associated with immune response and amino acid metabolism are over-represented in agreement with the data presented in [26]. Nevertheless, this analysis also points to the overexpression in aged fly heads of the 5 imaginal disc growth factors (Idgf1 to 5). These factors have been shown to interact with insulin to control growth [37]. Our data suggest that these secreted factors may play an unexpected role in the aging process.

In summary, besides the transcriptional modifications observed in the other body parts (mitochondria and stress responsive genes) and changes linked with reproduction, the aging signature in Drosophila head is mainly characterized by a large but selective downregulation of genes involved in synaptic transmission at different levels.

## Discussion

In this paper we have presented for the first time an integrated comparison of transcriptome modifications in different Drosophila body parts during the aging process. Since the RNA samples of body parts originated from the same batches of flies, our approach eliminates any biases that might be encountered when comparing microarray data from separate experiments (differences in rearing conditions or age, in genetic background or in extraction methods). It thus makes it possible to compare reliably age related transcriptional changes in the different body parts. In addition, we have shown that, in some cases, this approach can detect regionalized transcriptional changes that are otherwise diluted in whole body studies. In a Gene Ontology analysis, we ascertained that the head transcripts were enriched in genes involved in eye structure, neuronal or glial functions, while the thorax transcripts were enriched in genes involved in muscle structure or function. In addition, GFP or lacZ labelling from enhancer trap GAL4 lines associated with body part enriched genes show that our data can also provide a reliable expression assignment for genes of unknown function. Thus it will be a useful general tool for further studies of Drosophila.

When we first analyzed data from young and old whole flies and performed a comparison with previous data obtained with males [27,31] or females [28], we noted a good agreement with the two experiments on males, but a poorer correlation with the data for females. This was particularly striking for the genes downregulated in aged flies. Using the detailed microarray analysis of sex biased genes of Parisi et al. [29], we could assign about 50% of the downregulated genes in each sex to gonad enriched genes. This suggests that reproductive senescence accounts for a major part of age-associated transcriptome downregulation. Interestingly, these genes include several genes whose rate of change was described as reduced under caloric restriction treatment [28]. In addition transcriptional downregulation of genes linked with reproduction is not limited to the gonad, since many genes from this category (especially members of the Acp and Mst families) display the same behaviour in the head. Their role in the head, especially during the aging process, thus clearly deserves further attention.

At the whole body level, we observed age-related alterations in gene expression that confirmed previous reports: the metabolism of the organism seems to be severely affected, since genes linked with oxydative phosphorylation, TCA cycle, ATP production are downregulated, while those involved in purine biosynthesis are upregulated. Nevertheless, even for these general processes, our analysis reveals the existence of regional differences in transcriptional changes. We observed that, in some cases (e.g. TCA cycle genes in Additional file 5), these differences correlate to differences in expression levels in the different body parts. Moreover, different age-related gene modulations can be observed within a single process. For instance, in mitochondrial respiration, which has been shown to be altered with age [38], the proportion of downregulated genes is lower in complex I (25/36, 69%) than in complex II (6/7, 86%), III (10/11, 91%) or IV (11/13, 85%) and, for most of them, repression is significantly reduced in the thorax only. This suggests that the consequences of aging on metabolism may differ depending on the tissue.

One major reason for such differences may reside in tissue-specific mitochondrial impairment, which could be related to the level of cellular stress experienced during aging. Our data strongly suggest that mitochondrial function is more strongly impaired with age within the thorax, where energy requirement and metabolic levels are maximal during lifespan. Interestingly, biochemical data also point to a decline in mitochondrial respiration and electron transport in the thorax of old flies [38]. Moreover, a direct alteration of mitochondria in flight muscles of old flies has also been recently observed [39]. Considering our results, it would be of great interest to investigate whether this alteration is limited to muscles only or whether it also occurs in other tissues. Increased transcriptional impairment of genes encoding components of mitochondria correlates with other indicators of increased stress in thorax cells, such as the upregulation of the stress responsive transcription factor *jra *(*dJun*) with many of its targets and the upregulation of proteasome subunits, which was also observed in flies submitted to oxidative stress [30]. Our findings are also in agreement with previous data that showed that Hsp70 is induced preferentially in thorax in aging flies in correlation with the level of oxidative stress [40]. The order of causality between these events needs to be determined by additional experiments. Our data, however, suggest that severe impairments in thorax muscles may be one important cause of death. Similar features have recently been reported for the nematode [41].

An important finding of this study is the upregulation of many proteasome subunits in the Drosophila aging thorax that was not detected at the whole body level. In mammals, transcriptional modification of genes encoding proteasome subunits during aging has been controversial (see [42]) but the most recent microarray studies with complete genome coverage and high statistical power indicate that at least some proteasome subunits (PSMD5, PSMB4, rPA28, PSMD4, PSMB1 and a PSMC6 ortholog) are upregulated in aging muscle [23,24]. With the exception of the PSMD5 ortholog (CG12096), all the Drosophila genes coding for orthologs of these subunits (CG12000, REG, Pros54, Pros26 and Rpt4) are upregulated in aged Drosophila muscles. In addition, l(2)05070, encoding the ortholog of the inflammation inducible proteasome subunits LMP2 and LMP7 that have been shown to be over-expressed at the protein level in rat aged muscles [42], is also upregulated in aged Drosophila muscles. Together these data suggest that the mechanisms of regulation of proteasome subunits during the aging process are conserved from Drosophila to mammals. In rats, it has been proposed that such modifications may lead to an alteration in the degradation efficiency of the proteasome, which could participate in sarcopenia in aging rats [42]. Considering the possible conservation of regulation between species that we observed it would be interesting to address these hypotheses in Drosophila for which powerful genetic tools are available. The origin of the age-related transcriptional misregulation of proteasome subunits is still unknown. Nevertheless, its correlation with the level of mitochondrial impairment both in aging flies and in flies submitted to paraquat treatment (i.e., expected to inhibit the mitochondrial respiratory complex 1) may indicate a causal relationship between these events. It has also been recently proposed, on the basis of changes in caspase activity, that age-related increased apoptosis may play a role in Drosophila muscle degeneration [43]. Our data show that the caspase Damm and several proapoptotic factors (Cas, CG17765, CG8400, CG12384, CG12876) are upregulated in aging thorax. Thus the relative contribution of proteasome activation and apoptosis to age-related Drosophila muscle degeneration warrants further investigation.

Another major consequence of aging in flies revealed by this study is the transcriptional impairment of many genes in the head involved in synaptic transmission at different levels : genes involved in neurotransmitter metabolism, neurotransmitter secretion or neurotransmitter receptors are downregulated with age. Importantly, both inhitory and excitatory synapses seem to be affected during the aging process. Our data point to age-related modifications in cholinergic, dopaminergic, glutamatergic and GABAergic neuronal transmission. A combination of additional electrophysiological and behavioural measurements are clearly required to extend this data and identify the physiological consequences of the observed transcriptional modifications.

Besides age-related tissue specific transcriptome changes, our analysis also identified a further complexity in the relationship between aging and certain biological processes such as oxidative stress response and immune response. Indeed, although common gene upregulations may suggest that such processes participate in the aging process, as previously described, we show that their complete transcriptional signatures, as reported in separate experiments, differ significantly from those observed during the aging process. This is particularly striking for genes downregulated after paraquat stress or immune challenge, since, to a large extent, they do not present similar transcriptional variations in our aged flies. In the first case, since a strong correlation between the responses to aging and to hyperoxia is well documented [27], this different signature may be linked to the specificity of the paraquat stress, which is known to act through inhibition of mitochondrial complex I but could also interfere with NO homeostasis [44].

In the case of immune responsive genes, the same trend, observed on 40 day old flies in this study, can be also observed for 61 day old male flies according to the data of Landis et al.. Age related impairment in immunity has been reported recently [45], which results in a prolonged response following immune challenge. In addition, complex crosstalks between AP1 and NFκB signaling pathways involving histone deacetylase have been shown to modulate the kinetics of immune peptide production [46,47]. Our data point to a severe age related impairment of gene repression during immune response. It is tempting to speculate that age-related chromatin modifications may lead simultaneously to the release of NFκB control by AP1 and to the wider release of immune stress responsive gene repression during aging that we observed. Further work is needed to address these issues.

## Conclusion

We have provided in this paper an integrated description of transcriptional changes that occur in Drosophila body parts during aging. The tissue-specific variations that we have described point to large regional differences in the pathways and biological processes involved in aging. It emphasizes the need for a more complete description of transcriptional changes in longevity mutants. Our data open the way to the the identification of tissue specific age related molecular markers that will be useful for this task.

## Methods

### Stocks and collection of fly tissues

After emergence Canton S wild type males from a Canton S strain were placed in groups of 30 in vials and maintained at 26°C with a 12:12 light-darkness alternation. They were transferred to new vials every two days and collected at appropriate times (3 and 40 days) for tissue collection.

For each array 1300 males were frozen in liquid nitrogen and 300 of them were directly stored for subsequent RNA extraction of whole body flies. The heads of the remaining 1000 males were collected by fly vortexing and sieving through 710 μm and 350 μm meshes. The remaining parts of the body were used for manual separation of the thorax from the abdomen on a dry ice layer. Independent batches of samples from separate experiments were used for replicate experiments. All fly manipulations were performed at the same stages of the 12:12 light cycle to prevent any undesirable effects due to circadian variations.

### RNA sample preparation and data analysis

Total RNAs were purified by three rounds of Trizol reagent (GIBCO/BRL) extraction before precipitation. RNA quality was assessed using Agilent's Bioanalyser. cDNA were synthesized from 10 μg total RNA aliquots and biotin-labelled cRNA targets synthesized by using the BioArray high yield RNA transcript-labelling kit (Enzo Biochem) according to the manufacturer's instructions. Independent RNA pools were used for each array. Hybridizations on Drosgenome 1 Arrays (Affymetrix) and subsequent washing were performed on a GeneChip Fluidics Station according to the manufacturer's instructions before scanning on a GeneArray scanner. Three arrays were used for each condition with the exception of the 3 day heads for which 2 arrays were used.

### Data analysis

Together with our own data we processed simultaneously the raw data from [27] obtained on 10 and 61 day old male flies analyzed with the same Affymetrix chips, kindly provided by J. Tower. Such a procedure allows a more straightforward comparison of the two experiments on whole flies, since it rules out any bias due to differences in the analysis process. Extraction, normalization and computation of the expression indexes were performed using the RMA function of Bioconductor's affy package [48,49]. As already shown for other microarray data [50]we noticed that RMA tends to compress the fold change. We used a RMA FC threshold of 1.5 for statistical analysis that corresponds to a 1.69 FC threshold with a MAS5 treatment.

To increase the confidence level of our analysis an additional detection filtering was applied: for each probe set included in the analysis we required that at least one detection p-value provided by the MAS5 Affymetrix program in the different conditions (whole body, head or thorax) is lower than 0.1. This defined 8760 probesets which were used for further analysis.

The statistical significance of transcriptional variations was assessed using SAM software with a fold change (FC) threshold of 1.5 and a false detection rate (FDR) lower than 1% [51]. Under these conditions, we checked that, when our data were analyzed either alone or together with data from Landis et al., i) the same significant genes were selected and ii) that their fold changes were very similar. Thus the co-processing of both datasets did not skew the results.

Two different clusterings were performed on the data using the Boolean index (1: significantly upregulated, -1: significantly downregulated, 0: no significant change) provided by the SAM analysis in various two condition comparisons. In the first analysis we considered the enrichment in head or thorax compared to whole body at 3 and 40 days. To enhance the signification of the analysis we took into account for clustering only the probe sets that presented in a given body part the same type of variation for the two time points. This made it possible to reduce the original size of the Boolean space from 4^3 ^= 81 combinations to a cluster space of 12 indexes. The correspondance between the Boolean space and the cluster index is given in Additional file 6. In a second analysis age-related changes were studied for whole body, head and thorax through comparisons between the 3 day and the 40 day time points. The size of the original Boolean space (3^3 ^= 27) could be reduced to a cluster space of 14 indexes since some Boolean combinations correspond to none or very few probe sets (see Additional file 6 for the probe set number of each Boolean combination and the correspondance between the two spaces).

### Functional analysis

Information from the Gene Ontology (GO) database was combined with the Affymetrix data to investigate which classes are over- or under-represented in the dataset of stress responsive genes. Briefly, according to the Gene Ontology hierarchical structure, each probe set was assigned, when possible, to its original annotation and to the associated parent annotations. The number of probe sets for the different GO terms was computed for groups of probe sets defined according to different criteria (such as whole microarray probe sets, detected probe sets or probe sets belonging to a given cluster).

For each GO term G, the distribution between the group D of all the detected probe sets (N^G^_D _probe sets issued from a total of N_D_, probability P_G _= N^G^_D_/N_D_) and a group C of particular interest, such as a cluster (N^G^_C _probe sets issued from a total of N_C_) were compared. The hypothesis of equal distribution between these two groups would predict that, inside the N_C _probe sets of group C, N_C_*P_G _probe sets should be associated to the GO term. We computed the *p-value *P_N _for the null hypothesis of no association between the two distributions, with a hypergeometric distribution with N_C _tries, a probability P_G _and N^G^_C _successes. The p-values were corrected for multiple testing using the Benjamini Hochberg step-up procedure. Threshold values for P_N _helped to define the GO terms over- or under-represented in the group C.

### Microarray data comparisons

We compared our data with other microarray data from the following references: [27-30,32,34]. When the microarrays used in these studies were different from the Drosgenome 1 array used in our study we used a correspondance table between probe sets on each array based on the FlyBase ID number attributed to each probe set. We included in a Microsoft Access database the informations from these studies, including the clusterizations we had performed. For simplicity we used a numerical cluster coding for each study. Thus the character coded clusters of [Pletcher, 2002 #19] were transformed as follows: A→1, B→2, ..., P→16. Similarly, on the basis of the lists of probe sets provided in the aging/hyperoxia study of Landis et al. the following code was used: Old up→1, Old+O_2 _up→2, Old up+O_2 _down→3, Old down→4, Old+O_2 _down→5, Old down+O_2 _up→6, O_2 _up→7, O_2 _down→8. On the basis of this information, straightforward database requests generate cluster correlation tables between different experimental conditions.

### Gene expression analysis

NP GAL4 enhancer trap lines obtained from the DGRC (Kyoto, Japan) were crossed at 25°C to UAS-GFP lines for external visual examination of the progeny and to UAS-LacZ lines. The progeny from the latter cross was cryoprotected by overnight immersion in a 20% sucrose, 1X PBS solution before cryosectionning. Slides were then labelled by X-GAL staining according to standard protocol.

## Authors' contributions

FG participated in the design of the study, conducted the microarray experiments with the Affymetrix platform and performed the original normalization data treatment. CL conducted the βGal expression analysis and helped with the writing of the paper. VM participated in the design of the study, provided comments on the data analysis and helped with the writing of the paper. HT planned the study, analyzed the data and drafted the paper.

## Supplementary Material

Additional File 1 **Age response and body parts enrichment of DrosGenome1 probesets**. For each probe set *k *of the array (column 1) we calculated, for each comparison of interest between two experimental conditions C1 and C2, the mean ratio R_*k *_= < (S_c1_^*i *^/S_c2_^*j*^) > *i*,*j *where S_c1_^*i *^and S_c2_^*j *^denote the signal value for probe set *k *measured for the *i*^*th *^sample in the C1 condition and the *j*^*th *^sample respectively in the C2 condition. The standard error (SE) for each comparison is also reported. The index of detection (column 2) is put to 1 if at least one detection p-value obtained in the MAS5 Affymetrix program analysis in the different conditions (whole body, head or thorax) is lower than 0.1. In the sheet "clustered" the same mean ratio is reported for the probeset which have been identified as responsive either in one aging experiment (this study, [1,2]) or identified in body parts enrichment analysis (this study). Columns 6 to 10 contain the cluster index corresponding to the different analysis (see main text for details). Informations about the gene associated to each probeset is provided in columns 2 to 5. To facilitate visual inspection, we used a color code (red colors corresponding to upregulation, green colors to downregulation) with thresholds corresponding to fold changes of 2 (dark colors), 1.5 (medium) and 1.3 (light). Click here for file

Additional File 2 **Most prominent body parts enriched probesets**. The probesets enriched at least 4 times in head or 2 times in the thorax are listed with the associated gene informations (columns 2 to 6), the cluster index for the aging and tissue analysis (columns 7 and 8), the mean fold change compared to whole body at 3 days and 40 days (columns 9 and 10) and the mean of these two values (column 11). Click here for file

Additional File 3 **Functional analysis of age responsive and body parts enriched genes**. We analyzed the distribution in functional classes (as defined by the Gene Ontology (GO) database) of the genes selected by the SAM analysis (responsive genes) and compared it to the same distribution for all the genes significantly detected on our microarrays (analysed genes). We report here the number of analysed (Nref, column 4) and responsive genes (N, column 5) found inside these GO classes, for the different conditions in separate sheets. The number of expected genes (Nexpected, column 6) and the p-value (column 7) associated to the null hypothesis of no association with a hypergeometric distribution hypothesis is given for each class. The p-values corrected for multiple testing using the Benjamini Hochberg step-up procedure are given in column 8. The statistically significant under-represented classes are colored in orange while the over-represented classes are colored in green. **Sheets description:****WB_down, WB_up: **age downregulated or upregulated genes in whole body (this study). **head_down, head_up: **age downregulated or upregulated genes in head. **thorax_down, thorax_up: **age downregulated or upregulated genes in thorax. **triple_WB_down, triple_WB_up: **downregulated or upregulated genes in whole body in the three aging experiments (this study, [1,2]). **All_BP_down_cluster12, All_BP_up_cluster1: **downregulated or upregulated genes simultaneously in whole body, head and thorax. **Head_enriched, thorax enriched: **genes enriched either in head (clusters 1 to 4) or in thorax (clusters 1, 5 to 7). Click here for file

Additional File 4 **Common age responsive genes**. We report here the 112 probe sets which were identified as age responsive in the three aging experiments (this study, [1,2]). Informations about the gene is provided in columns 2 to 5, cluster indexes from these experiments in columns 7 to 10 and the mean values for the associated ratios of interest in columns 13 to 19. Additional data from [3] and a stress response experiment [4] are given in columns 11, 12 (cluster indexes) and columns 20 to 23 (fold change under stress conditions). Click here for file

Additional File 5 **Age response of genes belonging to different functional classes**. We report here the age response of genes belonging to some significant Gene Ontology classes. In each sheet are given: the identification of the GO class considered (columns 1, 2), Informations about the probe set and the gene associated (columns 3 to 6), cluster indexes from different analysis (columuns 7 to 10), the mean fold change during aging in whole body, head and thorax (column 11, 12 and 13 respectively), the enrichment in head (columns 14, 15) or thorax (columns 16, 17) compared to whole body. Click here for file

Additional File 6 **Boolean clustering**. We report here the correspondance between the final cluster index for aging response or body parts enrichment and the complete boolean coding which can be generated after SAM analysis. This step generates for each of the comparisons identified in line 1 a coding with three possible values: -1: significantly downregulated, 0: no change, 1: significantly regulated. This generates for the aging clustering a total of 3^3 ^= 27 boolean conditions and for the body parts clustering a total of 3^4 ^= 81 boolean conditions. To get a more readable clustering with a lower number of clusters, since the number of probe sets associated to many of these conditions is nul or very low, it is possible to regroup these boolean clusters to get the final cluster index reported in column 1. This index is used subsequently in all the analysis reported in this study. 1. GN Landis, D Abdueva, D Skvortsov, J Yang, BE Rabin, J Carrick, S Tavare, J Tower: **Similar gene expression patterns characterize aging and oxidative stress in Drosophila melanogaster**. *Proc Natl Acad Sci U S A *2004, **101**:7663–8. 2. SD Pletcher, SJ Macdonald, R Marguerie, U Certa, SC Stearns, DB Goldstein, L Partridge: **Genome-wide transcript profiles in aging and calorically restricted Drosophila melanogaster**. *Curr Biol *2002, **12**:712–23. 3. M Parisi, R Nuttall, P Edwards, J Minor, D Naiman, J Lu, M Doctolero, M Vainer, C Chan, J Malley, et al: **A survey of ovary-, testis-, and soma-biased gene expression in Drosophila melanogaster adults**. *Genome Biol *2004, **5**:R40. 4. F Girardot, V Monnier, H Tricoire: **Genome wide analysis of common and specific stress responses in adult drosophila melanogaster**. *BMC Genomics *2004, **5**:74. Click here for file
